# Analysis of PCR Kinetics inside a Microfluidic DNA Amplification System

**DOI:** 10.3390/mi9020048

**Published:** 2018-01-28

**Authors:** Jyh Jian Chen, Kun Tze Li

**Affiliations:** Department of Biomechatronics Engineering, National Pingtung University of Science and Technology, 1 Shuefu Road, Neipu, Pingtung 91201, Taiwan; milk85214560@gmail.com

**Keywords:** continuous-flow, polymerase chain reaction, DNA kinetics, kinetic equations

## Abstract

In order to analyze the DNA amplification numerically with integration of the DNA kinetics, three-dimensional simulations, including flow and thermal fields, and one-dimensional polymerase chain reaction (PCR) kinetics are presented. The simulated results are compared with experimental data that have been applied to the operation of a continuous-flow PCR device. Microchannels fabricated by Micro Electro-Mechanical Systems (MEMS) technologies are shown. Comprehensive simulations of the flow and thermal fields and experiments measuring temperatures during thermal cycling are presented first. The resultant velocity and temperature profiles from the simulations are introduced to the mathematical models of PCR kinetics. Then kinetic equations are utilized to determine the evolution of the species concentrations inside the DNA mixture along the microchannel. The exponential growth of the double-stranded DNA concentration is investigated numerically with the various operational parameters during PCR. Next a 190-bp segment of *Bartonella* DNA is amplified to evaluate the PCR performance. The trends of the experimental results and numerical data regarding the DNA amplification are similar. The unique architecture built in this study can be applied to a low-cost portable PCR system in the future.

## 1. Introduction

Since the polymerase chain reaction (PCR) was invented in the early 1980s, PCR has become one of the most important techniques in gene analysis, identification of infectious diseases, clinical diagnostics, and so on [1]. PCR is a procedure that exponentially amplifies a small amount of deoxyribonucleic acid (DNA) fragments into a lot of sample products during several thermal cycles. A PCR process goes through three major steps: double-stranded DNA is separated into two single strands at about 95 °C during denaturation; then primers bind specifically to their complementary site of the single-stranded DNA at about 55 °C during annealing; and DNA strands are extended by a thermostable DNA polymerase at about 72 °C during the extension. These steps complete one PCR cycle, and ideally each piece of DNA in the mixture is duplicated. By carrying out the three steps, the DNA concentration can be increased dramatically and then the DNA mixture can be analyzed through a commercial detector for the duration of PCR.

Nowadays PCR is mostly performed in a thermal cycler. Due to the large thermal mass of a commercial thermal cycler, the complete process usually takes more than 1 h. In order to reduce the processing time, miniaturized PCR systems have attracted much attention and accelerated the integration with the sample preparation, the DNA amplification, and the product detection into nucleic acid amplification testing systems since the 2000s. The small-scale thermocycling devices can be categorized into two groups: a chamber type and a continuous-flow type. These devices shorten the PCR reaction time from hours to minutes and have been reported for decades [2].

For a continuous-flow PCR (CFPCR) device, the reaction mixture moves through several different temperature regions in a thin channel or a tube instead of cycling the temperature of the whole reaction chamber for a chamber-type PCR device. The reaction time is the sum of each transition time and each residence time. The transition time is the time period for the sample traveling between two adjacent temperature regions. The residence time is the amount of time for a sample exposed to each temperature region to continue each PCR step. The transition time, the residence time, and the number of thermal cycles are determined by the flow rate of the DNA mixture, the arrangement of different temperature regions, and the geometrical pattern of the microchannel or the tube [3]. It has the benefit of the rapid thermal cycling by reducing the spacing between adjacent temperature regions and increasing the moving speed of the DNA mixture.

There are three main types of CFPCR reactors: unidirectional, closed-loop, and oscillatory reactors. Each has its own characteristic advantages [4]. In the unidirectional reactor, only an external device is needed to push the DNA sample through a single channel. The sample flows into the channel, experiences several temperature regions repeatedly, exits from the outlet, and completes the amplification process [5]. A self-propelled CFPCR in a microfluidic device that requires no external pump to drive the flow was also proposed. The PCR solution was dropped onto the inlet and autonomously transported by a capillary force [6]. For the unidirectional PCR device, no auxiliary devices such as valves and movable parts are needed. A home-made syringe pump [4] or reaction chip made of the polymer material [7] that is utilized in the unidirectional devices can greatly reduce the total cost of the system.

Each PCR step requires a specific temperature, so it is common to provide three temperature regions in the unidirectional PCR system [8,9,10]. When the DNA sample has reached the requisite PCR temperatures, the denaturation and annealing reactions happen within a very short period, and the extension rate is on the order of 60–100 bases per second [11]. A previous study also showed that the extension step could occur during the transition between the annealing and denaturation temperatures and no holding time was required [12]. This means that three distinct temperature regions arranged inside the CFPCR device are not necessary. In order to reduce the effort of the system integration and the cost of the device, some researchers have reduced the number of heaters to two while still obtaining the desired PCR thermal profile inside the CFPCR system [13,14,15]. A thermal system comprised of a heater and a heat sink was also designed to generate high- and low-temperature regions within the PCR device [16]. Furthermore, a CFPCR device employing only one heater was proposed. The geometry of the device was determined by proper simulations and the thermal control process for CFPCR was simplified [17].

Practically, the extent of PCR is related to the reaction rates of the kinetic equations inside the PCR mixture, which are functions of the temperatures and concentrations of reactants and enzyme [18]. Therefore, some researchers have developed mathematical models of PCR kinetics to investigate the mechanisms of PCR and calculate the DNA concentrations during reactions. Athavale et al. [19] performed a numerical simulation for PCR chemistry to evaluate the sample temperature and the concentrations of some chemical species in an oscillatory PCR reactor. A total of five major chemical species were assumed to control PCR. Mehra and Hu [20] mathematically analyzed the kinetics of each PCR step and investigated the effect of various kinetic parameters and operation conditions on the amplification efficiency. Priye et al. [21] presented the PCR process in a Rayleigh–Bénard convection cell. The PCR kinetics was incorporated into the flow model to obtain the time-dependent concentration profiles of some individual species. Papadopoulos et al. [22] proposed a comparison of the CFPCR and the chamber-type PCR devices through a computational investigation. The implementation of PCR kinetics to evaluate the performance of DNA amplification was shown.

To construct a CFPCR chip employing one heater or two heaters, the extension regions or the annealing regions are generated by the thermal gradients between the high-temperature and low-temperature regions. In previous studies, the temperature distributions inside the reaction regions were demonstrated numerically and/or experimentally and utilized to ensure that the requisite temperatures for PCR were existed [23,24]. In addition, by the rearrangement and enlargement of the channel geometry at the extension region, the residence time during the extension step can be properly extended to complete PCR [25]. However, the required thermal fields for PCR and the adequate residence time of each PCR step are not enough to connect with the successful PCR process. To understand the DNA amplification in a unidirectional CFPCR device, Wang and Wang [26] numerically studied the flowing effects on the DNA amplification with a simplified temperature distribution along the microchannel.

In our previous study [4], numerical simulations and thermal experiments have shown that the temperature distribution in the five-temperature-region (i.e., annealing, extension, denaturation, extension, and annealing regions) CFPCR chip was appropriate for DNA amplification. By utilizing one heater and two heat pipes to create the denaturation region and the annealing regions, efficient amplifications of DNA segments were proved to occur in our prior device. The main contribution of the current work is to fabricate a modified system and test the PCR performance of the one-heater CFPCR device by incorporating with one Peltier element. The unique architecture utilized in this flow-through PCR device can be successfully applied to a low-cost PCR system. In order to analyze the DNA amplification numerically with integration of the DNA kinetics, three-dimensional steady simulations, including flow and thermal fields, and one-dimensional PCR kinetics are presented. The central temperatures and velocity profiles of the cross-sectional area along the channel are exploited to calculate the concentrations of some specific species in the mixture. The simulated results are compared with experimental data that have been applied to the operation of a CFPCR device. Finally, results show that a 190-bp segment of *Bartonella* DNA is amplified successfully in the DNA amplification system.

## 2. Theoretical Modeling and Numerical Method

The continuous-flow PCR device is modeled to assess the flow, temperature, and concentration distributions of the DNA mixture inside the microchannel. The physical problem is a microfluidic chip based on a single meandering channel passing repeatedly through several temperature regions. The device consists of a reaction polydimethylsiloxane (PDMS) channel chip, a cover chip made of glass, and two aluminum blocks in contact with a cartridge heater and a Peltier element. The channel chip is 40 mm long × 25 mm wide × 1.9 mm high, and the glass substrate is 65.5 mm long × 25 mm wide × 1.1 mm high. The denaturation (high-temperature) region is heated by the cartridge heater and arranged at the chip center and the annealing (low-temperature) regions are located at the two sides. The temperatures of two annealing regions are regulated by the Peltier element. The extension regions are generated by the thermal gradients between the denaturation and the annealing regions. A combined radiation and natural convection condition is assumed except for the area in contact with the aluminum blocks.

**Figure 1:**
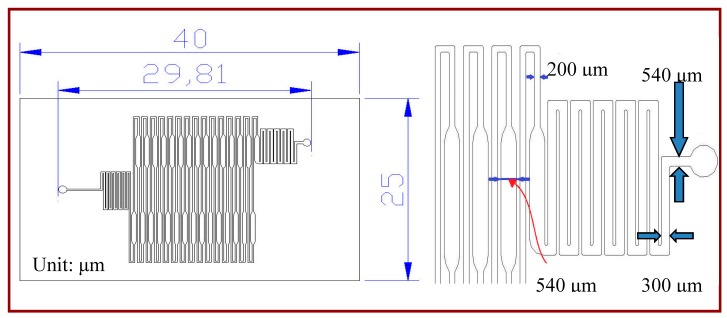
The schematic drawing of the continuous-flow polymerase chain reaction (CFPCR) chip.

A rectangular microchannel with a depth of 50 μm is utilized in the simulated model. The width of the microchannel is either gradually expanded from 200 μm to 540 μm and contracted back to 200 μm for the extension region, or 200 μm for the rest. Water is used as the required sample and the fluid properties are set as the physical and thermodynamic properties of water at 300 K. The sample is injected at the inlet at 300 K. Then it flows repeatedly through the denaturation (368 K) and annealing regions (328 K). Finally, it flows out of the outlet after passing the final extension region, as shown in Figure 1.

The governing equations consist of conservation of mass, momentum, and energy equations. The fluid flow is Newtonian, incompressible, and laminar. In symbolic notation, the steady state heat equation in the solid substrates can be expressed in vector form and shown below:

(1) $$\nabla^{2}T_{i}=0$$

where *T_i_* indicates the temperature of the material *i*, for *i* = 1 and 2. *T*_1_ and *T*_2_ indicate the temperatures of the materials of PDMS and glass, respectively. The steady state equations inside the microchannel can be expressed as follows:

(2) $$\nabla \cdot \vec{U}=0$$

(3) $$\vec{U}\cdot \nabla \vec{U}=\frac{-\nabla p}{\rho }+\nu \nabla^{2}\vec{U}$$

(4) $$\vec{U}\cdot \nabla T_{3}=\alpha_{3}\nabla^{2}T_{3}$$

where $\vec{U}$ is the fluid velocity vector in the channel; *p* indicates the pressure; and *ρ*, *ν*, *T*_3_, and *α*_3_ are the density, kinematic viscosity, temperature, and thermal diffusivity of the fluid, respectively.

The convection effect inside the microchannel on the temperature distributions within the chip is investigated in this study. In our study, the sample passes through three PCR steps repeatedly for 30 cycles to complete the PCR process. In order to simplify the simulation and reduce the computational time, the computational domain including only four of the thermal cycles is simulated and used to obtain the approximated condition.

The boundary conditions for the energy equation are thus as follows. The temperature regions in the device supported by the separate heating sources supply uniform temperature inputs.

(5) $$T_{2}=T_{w,k}$$

where *T_w_*_,*k*_ is the temperature of the heater and the Peltier element for *k* = 1 and 2, respectively. The three isothermal regions are a denaturation region and two annealing regions. The thermal contact resistances between two different materials are excluded, i.e., perfect contact surfaces are assumed. The temperature and the heat flux at the interface between two different materials, *m* and *l*, are set to be the same:

(6) $$T_{m}=T_{l}$$

and

(7) $$-k_{m}\frac{\partial T_{m}}{\partial n}=-k_{l}\frac{\partial T_{l}}{\partial n}$$

Thermal radiation energy transfer between the system and the ambient air is considered. The combined radiative and natural convective boundary conditions are used on all external surfaces except the surfaces in contact with the heater and the Peltier element.

(8) $$-k_{i}\frac{\partial T_{i}}{\partial n}=h(T_{i}-T_{\infty })+\varepsilon \sigma (T_{i}^{4}-T_{\infty }^{4})$$

where *k_i_* is the thermal conductivity of the material *i*, for *i* = 1 and 2; *h* is the convective heat transfer coefficient and is chosen as 5.005 W/m^2^·K [4]; *T*_∞_ is the ambient temperature; *ε* is the emissivity of the surface and is chosen as 1; and *σ* is the Stefan–Boltzmann constant, equal to 5.670367 × 10^−8^ W/m^2^·K^4^. For the microchannel, no-slip conditions are arranged at all the solid walls; a fixed-velocity and a preset temperature condition are set at the inlet; the boundary condition at the outlet is a fixed pressure.

PCR kinetics is employed for solving the species concentrations during the DNA amplification process. The overall process of PCR is complicated and a detailed chemistry model for PCR was proposed in the previous studies [20,27]. In our study, six major chemical species (i.e., double-stranded DNA molecule, single-stranded DNA molecule, enzyme, single-stranded primer molecule, single-stranded template–primer complex, and single-stranded template–primer–enzyme complex) are assumed to control the PCR process and five chemical reactions involved in the three steps of PCR are described as follows:

(1) Denaturation: The double-stranded DNA molecules, *D*, dissociate into two single strands, *S*. In addition, the high melting temperature causes thermal denaturation of enzyme, *E*, into inactivated enzyme, *E_i_*.
  (9) $$D\overset{k_{D}}{\underset{k_{-D}}{↔}}2S$$
  (10) $$E\overset{k_{E}}{\to }E_{i}$$
  where *k_D_* is the denaturation constant for melting of *D* at melting temperature; *k_−D_* is the renaturation constant for binding of *S* at melting temperature; *k_E_* is the enzyme inactivation constant. The arrow symbols “→” and “←” are used to denote net forward and backward reactions.
(2) Annealing: The single-stranded primer molecules, *P*, bind to *S* and form the single-stranded template–primer complexes, *SP*.
  (11) $$S+P\overset{k_{-SP}}{\underset{k_{SP}}{↔}}SP$$
  where *k_−SP_* is the annealing coefficient of *P* to *S*; *k_SP_* is the dissociation coefficient of *SP*.
(3) Extension: The polymerase enzyme, *E*, binding to *SP* to form the single-stranded template–primer–enzyme complexes, *E·SP*. Then the *E·SP* dissociates into *E* and *D* molecules at the beginning of the subsequent denaturation step.
  (12) $$E+SP\overset{k_{e}}{\underset{k_{-e}}{↔}}E\cdot SP$$
  (13) $$E\cdot SP\overset{k_{cat}}{\to }E+D$$
  where *k_e_* is the addition constant of *E* to *SP*; *k_−e_* is the dissociation coefficient of *E* from *E·SP* and *k_cat_* is the rate of nucleotide addition. From the previous study [21], increasing the complexity of the reaction model by addition of other kinetic reactions did not significantly alter the DNA amplification efficiency.

The kinetic reactions inside the continuous-flow PCR chip are modeled and the governing equations are presented as follows:

(14) $$\nu \frac{\partial [D]}{\partial s}=\frac{1}{2}k_{-D}{\left[S\right]}^{2}-k_{D}[D]+k_{cat}[E\cdot SP]$$

(15) $$\nu \frac{\partial [E]}{\partial s}=-k_{E}[E]+k_{-e}[E\cdot SP]-k_{e}[E][SP]+k_{cat}[E\cdot SP]$$

(16) $$\nu \frac{\partial [S]}{\partial s}=-k_{-D}{\left[S\right]}^{2}+2k_{D}[D]-k_{-SP}[S][P]+k_{SP}[SP]$$

(17) $$\nu \frac{\partial [P]}{\partial s}=-k_{-SP}[S][P]+k_{SP}[SP]$$

(18) $$\nu \frac{\partial [SP]}{\partial s}=k_{-SP}[S][P]-k_{SP}[SP]+k_{-e}[E\cdot SP]-k_{e}[E][SP]$$

(19) $$\nu \frac{\partial [E\cdot SP]}{\partial s}=-k_{-e}[E\cdot SP]+k_{e}[E][SP]-k_{cat}[E\cdot SP]$$

where *s* is the location along the central line inside the microchannel; *v* is the speed at the location *s*; and [*D*], [*E*], [*S*], [*P*], [*SP*], and [*E·SP*] mean the concentrations of double-stranded DNA molecules, enzyme, single-stranded DNA molecules, single-stranded primer molecules, single-stranded template–primer complexes and single-stranded template–primer–enzyme complexes, respectively. Reaction parameters in the present numerical simulation are listed in Table 1 and values reported in the literature are used [18,19,20,21,22,26]. *L* is the length of a DNA molecule. The rate constants in denaturation and annealing steps have a very sharp transition where the rates change from minimum (maximum) values to maximum (minimum) values. The rate constant for the extension step has a sharp peak at a reference temperature.

The commercial software CFD-ACE+^TM^ (version 2006, ESI Group, Paris, France), which uses a finite volume approach, is used to examine the three-dimensional steady velocity and temperature distributions in the continuous-flow PCR device, and the detailed simulation method was described in our previous study [4]. In order to achieve grid independence of the numerical results, several grid densities are investigated by comparing flow and thermal fields at various steps and the grid density is optimized for the accuracy and the speed of the simulation.

The numerical technique employed to solve the set of the governing equations of the kinetic reactions is the finite difference method. The central difference for the spatial derivatives in these equations is used to allow for yielding more accurate results than those from the forward or backward difference. The grid systems have been also checked to ensure grid-independent results.

## 3. Experimental Method

**Figure 2:**
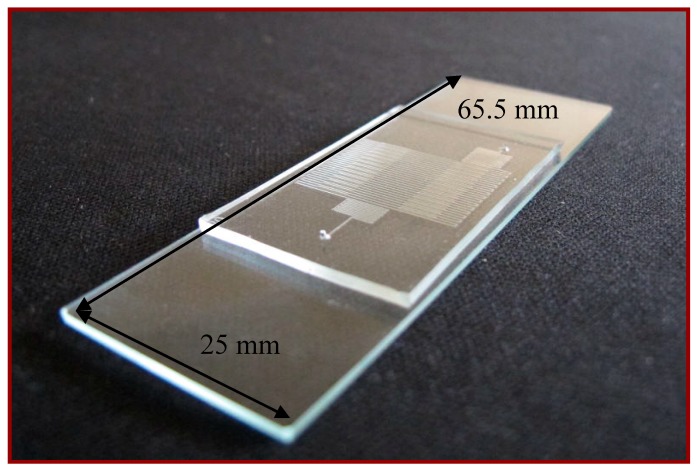
The PCR chip consists of a polydimethylsiloxane (PDMS) and a glass layer.

The microfluidic device consists of three subsystems including a PDMS-glass bonding chip, a home-made syringe pump, and a thermal control system. A serpentine channel with various widths meandering inside the PDMS chip is used for the DNA mixture to be pumped through. The comprehensive fabrication process of the PDMS-glass bonding chip has been depicted in our earlier work [3]. The PCR chip consists of a PDMS and a glass layer with thicknesses of 1.9 and 1.1 mm, respectively, as shown in Figure 2. The 30-loop channel of 50 μm depth is 200 μm in width, except for the extension region with a maximum width of 540 μm. The reactor channel of the chip is replaced after use.

The mixture is injected into the channel through the inlet and is driven by the syringe pump. After a designated number of thermal cycles, the reagent is taken out of the channel for further analysis. In our previous work, a home-made syringe pump was built and used to force the DNA sample passing through the reaction channel [4]. It costs less than $400 and is successfully applied to a low-cost PCR system.

In our designed chip, the denaturation region heated by one heater is located at the center of the chip and the annealing regions supported by a Peltier element are set at the two sides of the chip. The extension regions can be created by lateral heat conduction from the denaturation region to the annealing regions. A PCR thermal cycle is completed when the reagent leaves the extension region. The symmetric management creates the five reaction regions in the chip. The five-temperature-region design requires only one half-loop per PCR cycle in contrast to the conventional three-region design. The total chip volume can be greatly reduced.

When performing PCR, the PCR chip is attached tightly to the top-side of the aluminum blocks. The aluminum heating blocks and the PDMS-based chip are assembled and fixed onto a poly(methyl methacrylate) (PMMA) housing. The thermal control modules are designed to be detachable from the channel chip and to be reused. The chip temperature at the denaturation region during the operation is held by using the cartridge heater under a home-made proportional-integral-derivative (PID) controller. The temperature sensor, DS18B20, mounted onto the aluminum heating block is utilized to supply temperature feedback.

**Figure 3:**
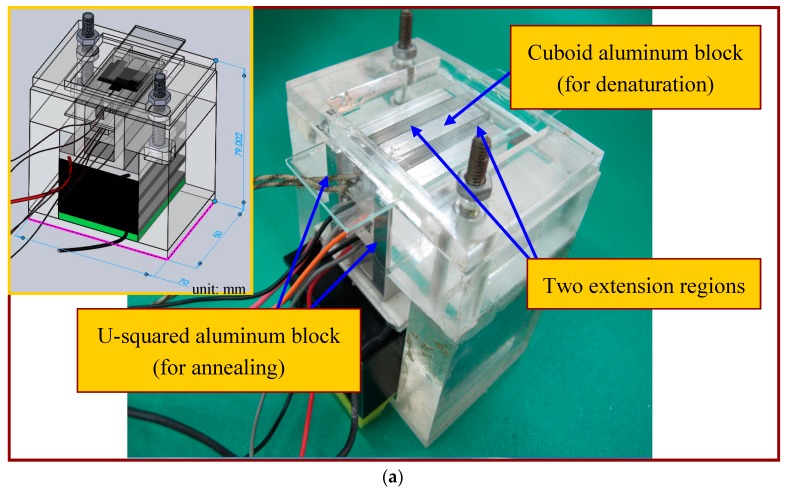

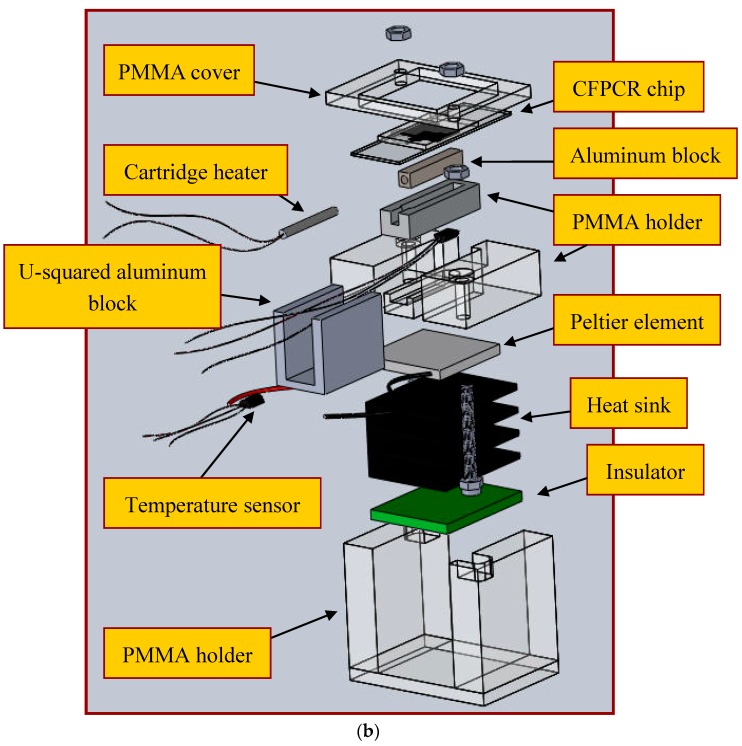
(**a**) The assembled CFPCR device; (**b**) an exploded view of the device.

The annealing temperature is controlled by the contacted Peltier element module and the sensor through a similar thermal control program for the cartridge heater. An aluminum block of a U-squared shape is put in contact with a Peltier element and a cooling fan is placed under the CFPCR chip to achieve the required temperature at the annealing regions. The block is in contact with the chip via some thermal conductive adhesive. The advantages of a Peltier element include: either heat or cool depending upon the polarity of the applied power, control temperatures to better than ±0.1 °C. The temperature difference of the block surface at three measured points is about ±1 °C. Figure 3a,b shows the assembled CFPCR device and the exploded view drawing of the device respectively. Compared with our previous study [4], two 6 mm-diameter heat pipes were pressed flat and placed in contact with the annealing regions of the chip. Attaching a fan to a portion of the heat pipe makes the heat pipe cool to the specific temperature for annealing. However, the optimal annealing temperature depends on the GC content of the primers, and is somewhere between 318 K and 338 K. It is impossible to heat up the region to the requested annealing temperature by placing the heat pipe in contact with a cooling fan when different kinds of DNA templates and primers are used. So it is more flexible utilizing the Peltier element than the heat pipe in the CFPCR chip.

To reduce the binding of biomolecules on the PDMS surface, the microchannel walls are treated with polysorbate 20 (Tween 20). Besides, mineral oil is introduced before the DNA mixture to suppress the formation of air bubbles, which tend to appear when performing the denaturation step [3].

Cat scratch disease (CSD) is the most frequent clinical manifestation of *Bartonella* infections in immunocompetent patients. Recently, several PCR-based assays have been developed for detection of *Bartonella* DNA in clinical samples. Evaluation of infected tissue or blood using PCR has been shown to be an effective tool for diagnosing *Bartonella* infection [28]. On the PCR chip and commercial PCR machine (MJ Mini™ 48-well Personal Thermal Cycler, Bio-Rad Laboratories, Inc., Hercules, CA, USA), a 190-bp segment of *Bartonella* DNA is amplified to evaluate the performance of the DNA amplification. The forward and reverse primers comprise the following sequences: 5′-ACG AAA GTC TGA TGG AGC AAT A-3′ and 5′-ACG CCC AAT AAA TCC GTA TAA T-3′, respectively. The PCR mixture contains 2× reaction buffer, deoxynucleotide (dNTP) mixture (400 μM), MgCl_2_ (3 mM), the forward and reverse primers (0.5 μM), DNA polymerase from *Thermus* sp. (0.05 U/μL) and template DNA (0.1 μg/μL).

The off-chip PCR for the commercial thermocycler involves heating the mixture at 95 °C for 3 min to activate the polymerase and denature the initial DNA, followed by thermal conditions consisting of denaturing at 95 °C for 30 s, annealing at 55 °C for 30 s, and extension at 72 °C for 30 s. Upon completion of up to 30 thermal cycles, the chip is kept at 72 °C for 3 min for the final extension. The negative control experiment is conducted by replacing the template genomic DNA with the nuclease-free water.

After the PCR process is finished, the products are analyzed by an agarose gel electrophoresis (Mini-Sub Cell GT System, Bio-Rad). Each sample is loaded onto 2% of agarose gel (Certified Molecular Biology Agarose, Bio-Rad) and electrophoresed in 10× Tris/Boric Acid/EDTA (TBE) buffer. The gel is run for about 40 min at 120 V. After electrophoresis, the gel is stained with 10 mg/mL ethidium bromide solution (Bio-Rad) and imaged under ultraviolet (UV) illumination.

We design a PDMS-based PCR chip consisting of a serpentine microchannel to perform the PCR process. Ten microliters of PCR solution were introduced into the microchannel by a home-made syringe pump at a specific flow rate. Mineral oil used to suppress the bubble formation in the microchannel is first injected into the microchannel and then followed by the PCR solution. The PCR solution consists of 0.25 μL of each of the forward primer and reverse primer, 2.5 μL of nuclease-free water and 2 μL of template DNA. After 30 thermal cycles, the PCR product is taken out of the chamber for further analysis.

## 4. Results and Discussion

The microfluidic chip is exposed to one heater and one Peltier element in order to create several temperature regions for PCR. In the following sections, the influences of operational parameters, such as sample flow rates and temperature settings at the isothermal regions, on the temperature distributions and the concentration profiles along the channel are comprehensively studied. Then the temperature measurements are demonstrated to ensure the requested PCR temperatures inside the chip. Finally, the PCR experiments are carried out in the serpentine channel.

### 4.1. The System Design Concepts

In this study, three design concepts are used to lessen the chip volume and save system costs. (1) One cuboid aluminum block with a cartridge heater to create the high-temperature region (denaturation) at the center of the chip and one U-squared aluminum block with a Peltier element to construct the low-temperature regions (annealing) at the opposite sides of the chip are integrated. Only two thermal modules for accomplishing the required PCR temperatures inside the chip are utilized; (2) By lateral heat conduction from the thermal blocks for denaturation and annealing, two extension regions are created without further active heating. Both the construction of five temperature regions within a PCR chip and the reduction of the chip volume are achieved; (3) The channel width at the extension region is enlarged. Because of the enlarged channel width, the flow rate of the DNA mixture is decreased and the residence time during the extension step is prolonged. This ensures that there is enough time for DNA extension without a longer channel. The fluid properties are set to the physical and thermodynamic properties of water, which are thermal diffusivity, *α*, of 1.39 × 10^−7^ m^2^/s, thermal conductivity, *k*, of 0.613 W/ms and kinematic viscosity, *ν*, of 8.55 × 10^−7^ m^2^/s. Thermal conductivities of PDMS and glass are respectively 0.15 W/ms and 0.58 W/ms. Five temperature regions are located within the chip width of 25 mm. The three temperature regions at the central part (6 mm wide) and the opposite sides of the chip (6 mm wide) are at temperatures of 368 K and 328 K. The above values of the parameters are used unless noted otherwise.

**Figure 4:**
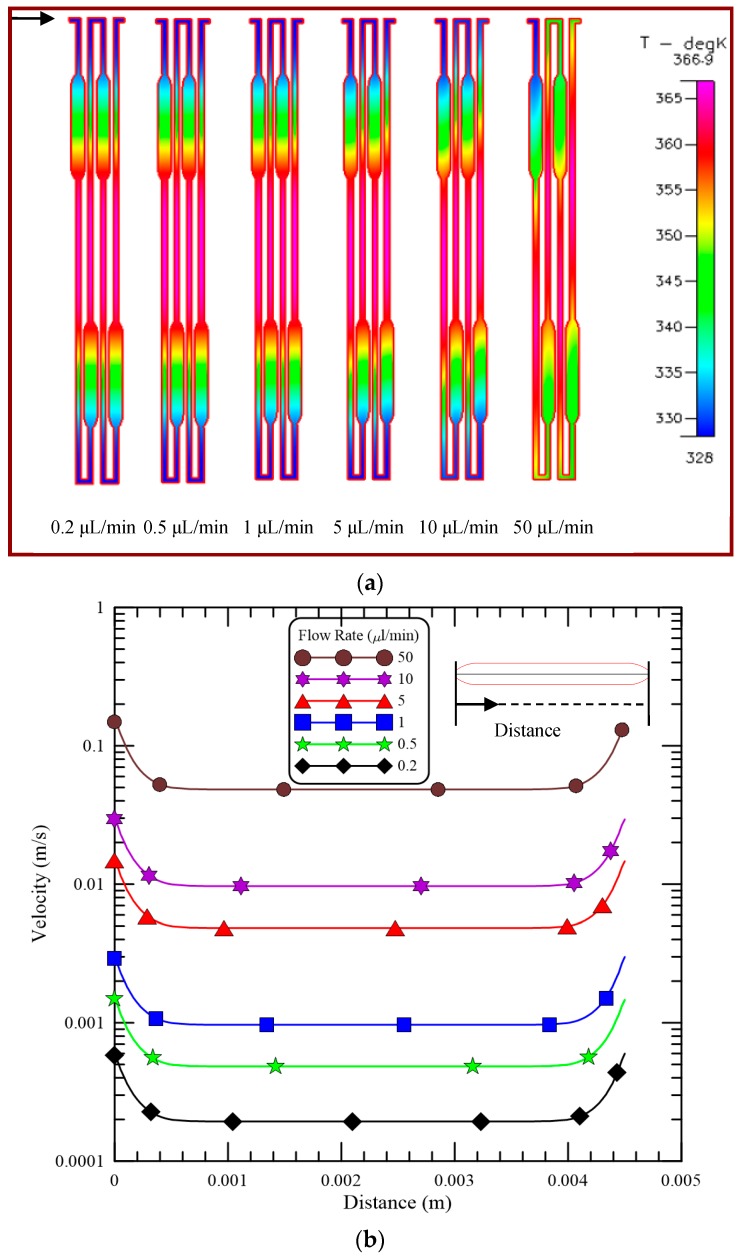
(**a**) The cross-sectional temperature distributions at the Y-half cut cross section of the channel and (**b**) the velocity profiles along the central line at the enlarged part of the channel for different flow rates.

For an efficient PCR process, the temperature variation of the DNA sample is one of the most important issues. The influences of various mixture flow rates on the cross-sectional temperature distributions at the Y-half cut cross section of the channel are plotted in Figure 4a. The arrow is given in Figure 4a to indicate the flow direction. The temperature distribution at this cross section can be treated as the temperature of the PCR mixture flowing along the microchannel. Flow rates of 0.2, 0.5, 1, 5, 10 and 50 μL/min are considered; these flow rates correspond to inlet flow velocities of 0.333, 0.833, 1.667, 8.333, 16.667 and 83.333 mm/s, respectively, and Reynolds number (Re) of 0.027, 0.067, 0.133, 0.667, 1.333, and 6.667, respectively. The temperature distributions show the three distinct temperature regions in the center area (for denaturation) and at the two sides of the chip (for annealing). The uniform temperature distributions at the regions of denaturation and annealing are obvious for our PDMS-glass bonding chip. The respective temperature is almost the same except for the flow rate of 50 μL/min and it changes gradually between three temperature regions. The temperature about 345 K can be created at the enlarged part of the channel for extension. The five-temperature region within a chip for PCR is apparent to be constructed. To accomplish the PCR temperatures inside the chip by using two different thermal regions is successful. For a laminar flow with a flow rate less than 5 μL/min, the fluid reaches the equilibrium temperature within a very short distance. The symmetric pattern of the temperature distribution with respect to the central region is observed and the heat convective effect inside the microchannel can be neglected. By increasing the flow rate, the heat convective effect inside the channel is enhanced. The distortion of the symmetric pattern of the temperature distribution is evident. This is because as the flow rate increases, there is less time for the mixture to achieve the desired PCR temperatures. We found that the flow rates of the DNA sample larger than 10 μL/min are not suitable for efficient PCR.

Figure 4b demonstrates the velocity profiles along the central line at the enlarged part of the channel for different flow rates. Simulated velocities are obtained along the downstream direction, which is shown in the top-right corner in Figure 4b. The velocities are almost the same except near the entrance and exit of the enlarged part of the channel for each flow rate. The flow velocity near the entrance and exit of the enlarged part of the channel is the fastest and almost equal to the velocity along the regular channel. The ratio of flow velocities in the enlarged and regular microchannels is about 3. The residence time during each PCR step can be calculated by the channel volume of the working region divided by the flow rate. This means the residence time during the extension step is extended without any doubt.

### 4.2. The Temperature Measurements

**Figure 5:**
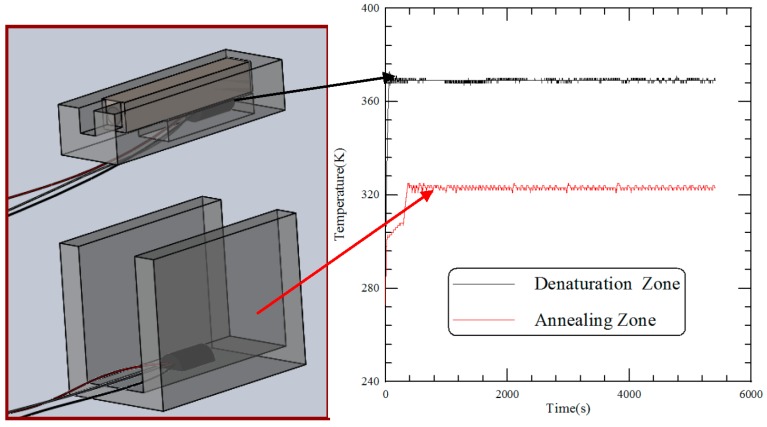
The transient temperature profiles of two thermal blocks.

Figure 5 shows the transient temperature profiles of two thermal blocks. After the heater and Peltier element are switched on, the measured temperatures of the thermal sensors attached under the heater block and onto the U-squared block are recorded. Results show that the rise times to reach the setting temperatures of 368 K and 323 K are less than 1 and 6 min. The time response for the Peltier element longer than that for the heater is partially due to the large thermal resistance across the thermoelectric cooler. The rise time specifies the time taken for the block temperature to rise from the ambient temperature to the setting temperature. In our device, it preserves a fast transition. The temporal variations of the block temperatures during the steady state are within 2 °C and 4 °C at the aluminum blocks with the heater and Peltier element, which fall in the allowable range.

**Figure 6:**
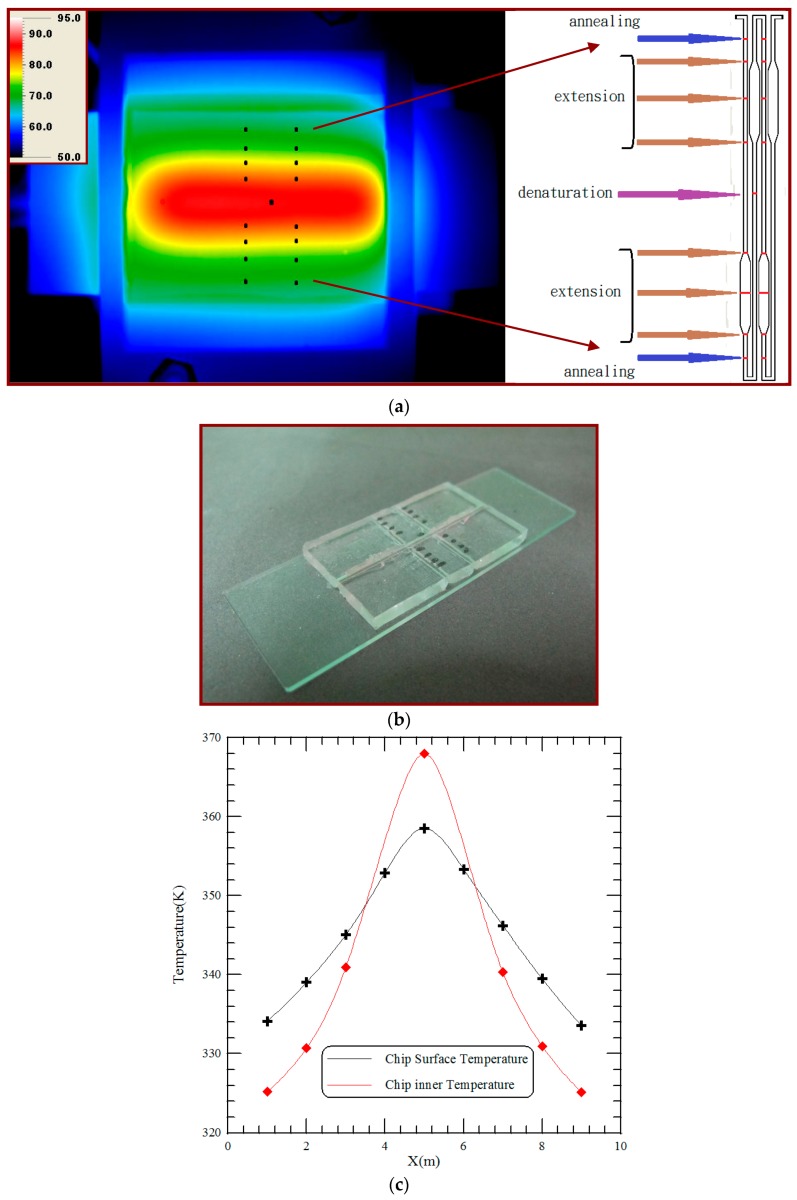
(**a**) The infrared (IR) image of the chip surface of the CFPCR device; (**b**) the channel temperatures are measured using thermocouples that are inserted into the chip; (**c**) the average temperature profiles for the IR image and the channel measurement.

Temperature uniformity is one of the important issues that influence the efficiency of biological reactions. An infrared (IR) camera (TAS-G100EXD, Nippon Avionics Co., Ltd., Tokyo, Japan) is utilized to characterize the spatial temperature distribution across the surface of the PDMS-based chip and evaluate the performance of the thermal modules. After reaching a steady state temperature distribution, IR images of the PCR device are captured. Figure 6a displays three distinct regions represented the denaturation region at the central part and the annealing regions at the two sides of the chip. The five-temperature region is arranged to support the DNA amplification. The captured digital images are then analyzed to determine the temperature profiles across the chip surface. The average temperatures of two paths are calculated from the experimental results. The channel temperatures are measured using thermocouples that are inserted into the chip [4], placed in contact with the glass and connected to a data acquisition system (Model NI 9211, National Instruments, Austin, TX, USA), shown in Figure 6b. The dimension of the chip is the same as that of the chip used for PCR (shown in Figure 2). A computer receives the temperature signals through the NI 9211 interface and records the real-time temperature profiles. The average temperature profiles are illustrated in Figure 6c. The solid line with cross marked symbols indicates the temperatures measured by the infrared imager, and that with square marked symbols represents the temperatures measured by the thermocouples. Results show a certain temperature difference between the chip surface (measured by the IR imager) and the PCR mixture (measured by the thermocouples) is found.

The thermocouples are calibrated in a water bath. After reaching a steady state temperature, the temperatures of the thermocouples are recorded. The thermocouple wire and extension wire are supplied to meet special tolerances, 1.1 °C, of ASTM E 230 (Pentronic, Västervik, Sweden). For the NI 9211 measurement system, measurement sensitivity, which represents the smallest change in temperature that a sensor can detect for the data acquisition system, is less than 0.07 °C. The uncertainty of the temperature measured in our experiments is less than 2.2% within the temperature range of 50 °C to 95 °C.

**Figure 7:**
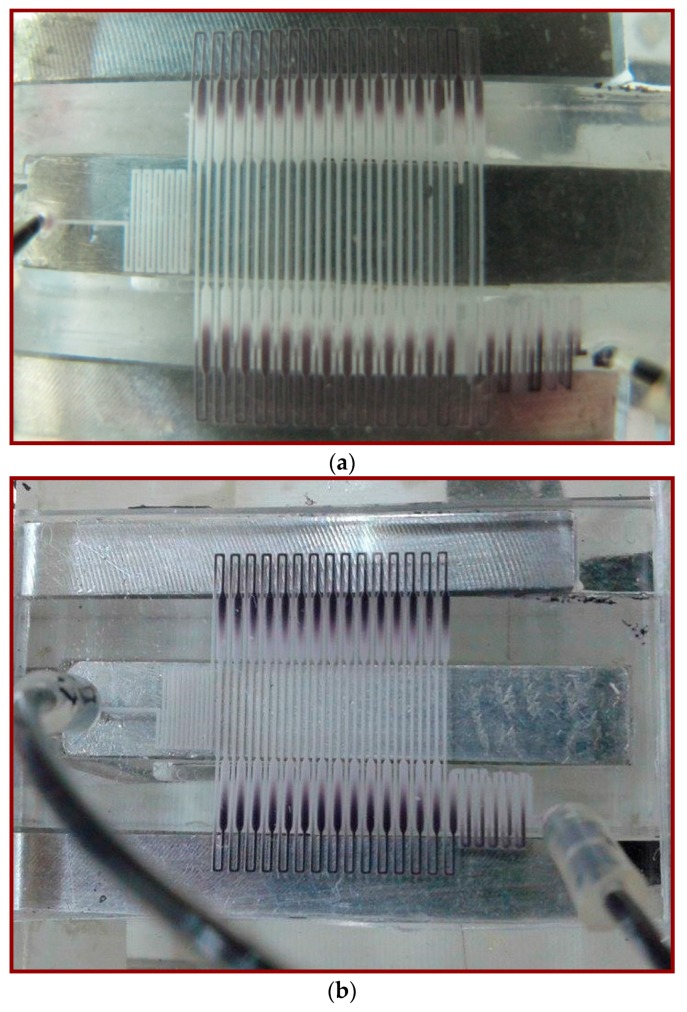
The temperature distribution visualized using a thermally sensitive dye. The fluid with dye is moving at the flow rate at (**a**) 0.2 μL/min and (**b**) 10 μL/min.

The sample transporting along the microchannel can also be visualized qualitatively using a thermally sensitive dye (TM-SL 70-3441, New Prismatic Enterprise Co., Ltd., Taipei, Taiwan). Using the thermally sensitive dye with an approximate transition temperature of 343 K, a visualization of the channel temperature distribution can be achieved. The dye becomes colorless when the temperature is greater than approximately 343 K and darker when the temperature is lower than roughly 343 K. Figure 7a shows that with a flow rate equal to 0.2 μL/min, the temperatures are within the PCR temperature limits. For a flow rate of 10 μL/min, neither the denaturation nor the annealing region temperatures are met for the setting temperatures of two thermal modules in Figure 7b. The dimension of the chip is the same as that of the chip used for PCR (shown in Figure 2).

### 4.3. DNA Kinetic Characteristics

Papadopoulos et al. [22] proposed an investigation into PCR amplification efficiency inside a CFPCR device through a numerical study. Regarding the specific boundary condition, the concentration distribution of double-stranded DNA molecules at the middle height of the microchannel was shown and the DNA amplification efficiency was demonstrated, too. In this section, DNA kinetic characteristics are analyzed by utilizing the PCR kinetics. The comprehensive analyses of the operational parameters on the concentration distributions of six species in the DNA mixture are investigated.

**Figure 8:**
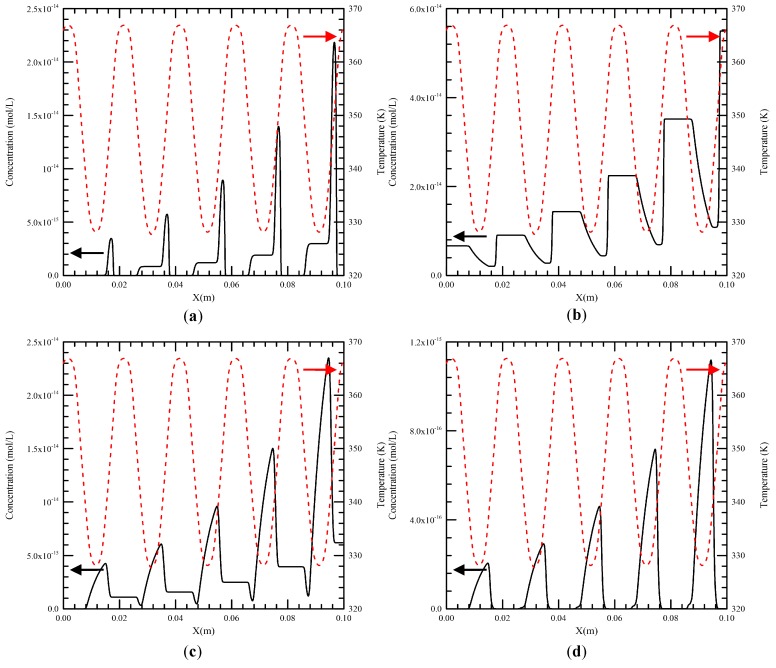
The concentration profiles of (**a**) double-stranded DNA molecules, (**b**) single-stranded DNA molecules, (**c**) single-stranded template–primer complexes and (**d**) single-stranded template–primer–enzyme complexes (i.e., [*D*], [*S*], [*SP*] and [*E·SP*]) along the central line of the channel.

From our previous work, the maximum temperature difference of the liquid flow at the cross section along the channel is less than 2 K [4]. So the temperature along the central line of the channel can be seen as the mixture temperature inside the channel. The fluid temperature and speed along the channel are introduced into the DNA kinetic equations (i.e., Equations (14)–(19)) to solve the concentration profiles of six species during the PCR process. The flow rate of the DNA mixture equal to 0.2 μL/min is used unless noted otherwise. The temperatures of the heater and the Peltier element are set at 368 K and 343 K. The length of the double stranded homologous DNA is assumed to be 200 bps. The initial concentration of each species in the PCR mixture is listed in Table 2. The concentration profiles of double-stranded DNA molecules, single-stranded DNA molecules, single-stranded template–primer complexes and single-stranded template–prime–-enzyme complexes (i.e., [*D*], [*S*], [*SP*] and [*E·SP*]) along the central line of the channel are presented in Figure 8a–d, respectively. These show the variation of the concentration profiles versus time for five cycles. The dashed line denotes the temperature profile. We can find in Figure 8a that the mixture enters the denaturation region, [*D*] decreases to a very small amount with an extremely fast speed and the dissociation of double-stranded DNA molecules is almost complete. Then [*D*] rises to a high value in the extension region. From Figure 8b, [*S*] starts with a specific value of initial concentration, grows to a high value (double in decreasing of [*D*]) in a very short period, declines in the annealing region, and then erupts when it enters the denaturation region. This local maximum [*S*] in one cycle is almost twice of the local maximum [*D*] in the former cycle. Not all single-stranded DNA molecules are bind to the primers in the annealing region and some of them enter the extension region. Corresponding plots for the single-stranded template–primer complexes are demonstrated in Figure 8c. With regard to [*SP*], it starts with a low concentration after denaturation, builds up when the mixture flows through the annealing region and is diminished in the extension region. This can be extended to generate single-stranded template–primer–enzyme complexes, shown in Figure 8d. Because of the large value of *k_e_*, that is the reaction constant of the binding of *E* and *SP*, [*E·SP*] follows up with [*SP*] simultaneously. After five thermal cycles, [*D*] increases exponentially as the mixture travels down the channel.

**Figure 9:**
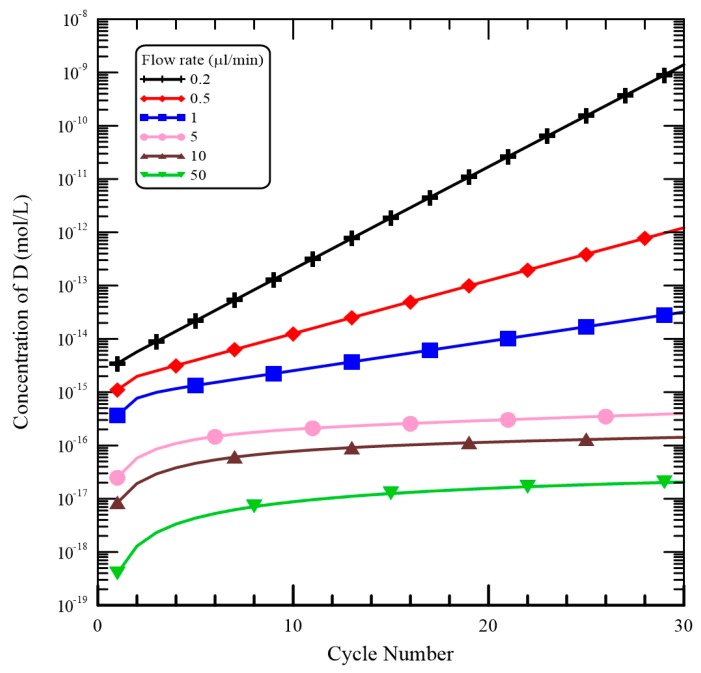
The influence of various flow rates on [*D*].

The mixture flow rate is important for the DNA mixture to achieve the requested temperatures in the CFPCR chip and it modulates the residence time of the DNA mixture in each reaction step. For the mixture with a flow rate less than 5 μL/min, the convective heat effect inside the microchannel can be neglected and the temperature distributions for various flow rates are similar. The effect of the flow rates on [*D*] shown in Figure 9 is noticeably more pronounced than the effect on temperature distribution. When the flow rate is equal to 0.2 μL/min, [*D*] increases dramatically as the number of thermal cycles became large. The higher the flow rate is, the slower the [*D*] increases. As the flow rate increases, all the reaction times in each step become short and the time for complete dissociation is not enough. From Figure 9, when the flow rate is larger than 5 μL/min, no longer does [*D*] almost increase after the cycle numbers larger than 10. So the amount of DNA product after 30 thermal cycles is still tiny and it almost cannot be detected.

**Figure 10:**
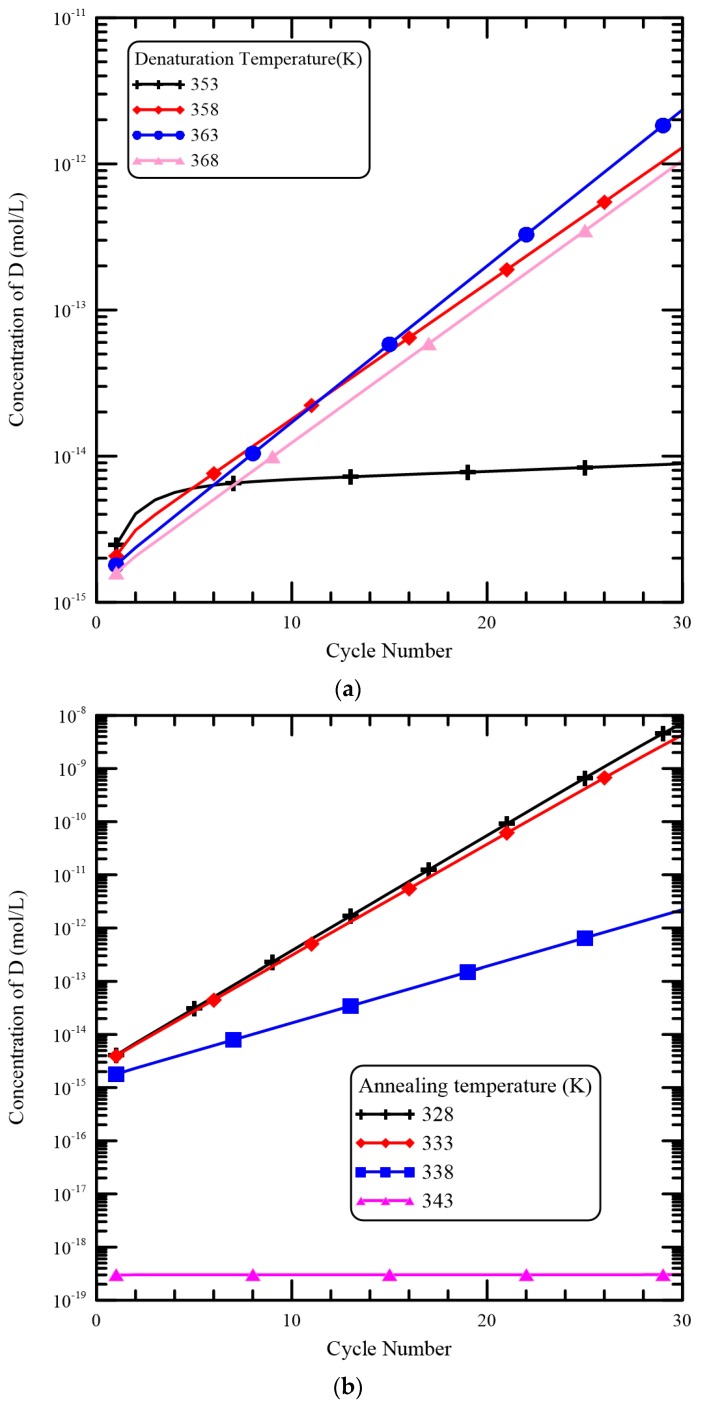
The influence of various temperatures of (**a**) the heater and (**b**) the Peltier element on the [*D*].

For the CFPCR device, the reaction mixture meanders through several different temperature regions in a microchannel. The temperatures of the thermal blocks define the temperatures of the reaction regions and have a profound impact on the temperatures distributions of the DNA mixture. The influence of various temperatures of the heater and the Peltier element on the [*D*] is illustrated in Figure 10. The flow rate is set to be 0.2 μL/min. From Figure 10a, the heater temperature is changed from 353 K to 368 K. The temperatures in the denaturation region shift to lower values as the heater temperature decreases so that the DNA solution will spend less time in high-temperature regions. However, the rate constants in the denaturation step have a very sharp transition over a 10 degree temperature change. For the constant values of the hyperbolic tangent functions used in *k_D_* and *k_−D_* are both 358, that is, the melting temperature for specific DNA strands is 358 K. This means the dissociation of *D* starts from 353 K to 363 K. As the heater temperature is equal to 353 K, the mixture temperature increases from 328 K (the temperature of the Peltier element) to about 353 K. Results show that the dissociation of *D* is not complete during the denaturation and the increasing of [*D*] in the extension region is not apparent. When the heater temperature is increased to be greater than 358 K, the mixture flows from a low-temperature region to the denaturation region and the mixture temperature is heated over 353 K. So the enhancement of [*D*] in the extension region is presented. Due to the fast dissociation process of *D*, the concentration profiles of *D* are similar for the cases of the heater temperature great than 358 K. Figure 10b demonstrates the results of various temperatures of the Peltier element on [*D*]. The temperature of the Peltier element is varied from 328 K to 343 K and the heater temperature is equal to 368 K. The constant values of the hyperbolic functions in the expression of *k_SP_* and *k_−SP_* are both 338, i.e., the melting temperature of a primer. Then single-stranded primer molecules can bind to single-stranded DNA molecules in the temperature range from 333 K to 343 K. When the temperatures of the Peltier element are 328 K and 333 K, the temperature of the annealing region is less than 333 K. As the mixture flows through the annealing region and the mixture temperature also decreases to a value of less than 333 K. Then *P* can bind to *S* successfully and *D* can exponentially increase in the extension region. Results show that the temperatures of the Peltier element are 328 K and 333 K and [*D*] is increased noticeably. As the temperature of the Peltier element equals 338 K, binding between *S* and *P* is not enough during the annealing step and the DNA amplification is not very successful. Finally, the temperature of the Peltier element is 343 K and almost no DNA amplification can be seen.

### 4.4. PCR Amplification in the Microfluidic System

**Figure 11:**
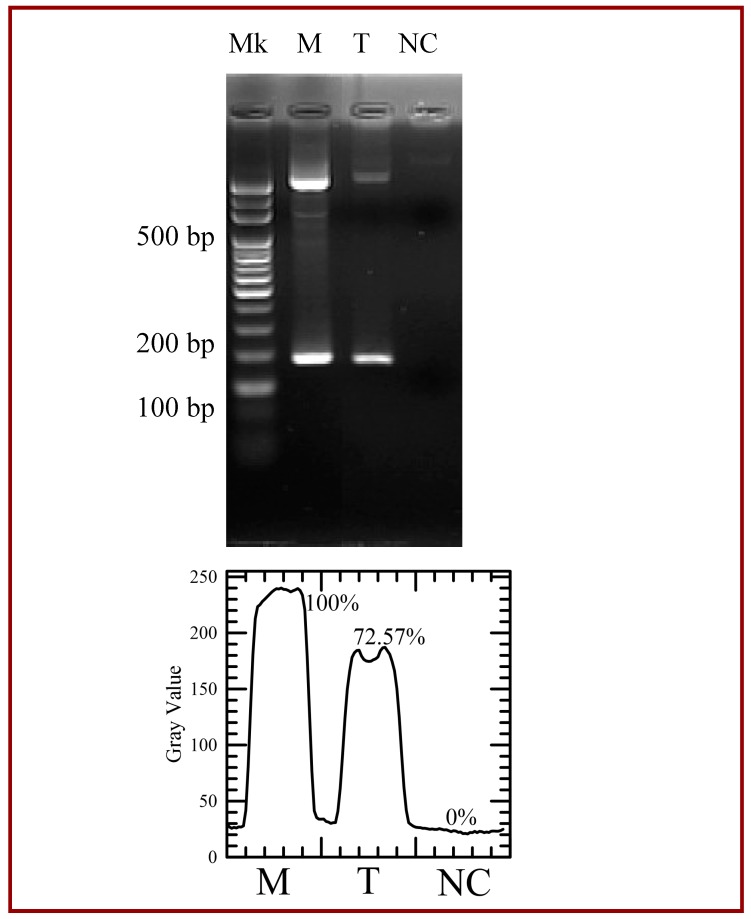
Agarose gel electrophoresis of the polymerase chain reaction (PCR) yield. The first lane (Lane Mk) indicates the DNA ladder. The 190-bp PCR product in the commercial PCR machine (Lane M) and in our device (Lane T). Lane NC indicates a mixture for a negative control. The gray intensities of PCR products are created by image analysis.

The performance of PCR amplification of the microfluidic system is compared to the results in the conventional PCR machine. The electrophoresis data of the PCR products are shown in Figure 11. The lane Mk in Figure 11 indicates the DNA ladder. From this gel electrophoresis analysis, the amplification products are successfully amplified in our device at a flow rate of 0.3 μL/min (lane T) and a PCR machine (lane M). A mixture containing all the reagents in the PCR mixture but the DNA template for a negative control is injected into the channel and collected after the total thermal cycles for analysis (lane NC). The 190-bp PCR products are observed and non-specific products are not found. Images are analyzed using the image processing software (ImageJ, Version 1.50b, National Institutes of Health (NIH), Bethesda, MD, USA). Intensity linescans of the fluorescence intensities are utilized to determine those intensities. The bottom of the figure also illustrates the grey intensities of PCR products by image analyses. It is noticed that the fluorescence intensity of the PCR product at lane T is smaller than that of the PCR products at lane M. The thermal cycling program for the commercial PCR machine includes each PCR step for 30 s and 35 cycles. The residence times for three PCR steps in the present device are less than 30 s. It might cause the inefficient reaction. However, the result is still obvious.

**Figure 12:**
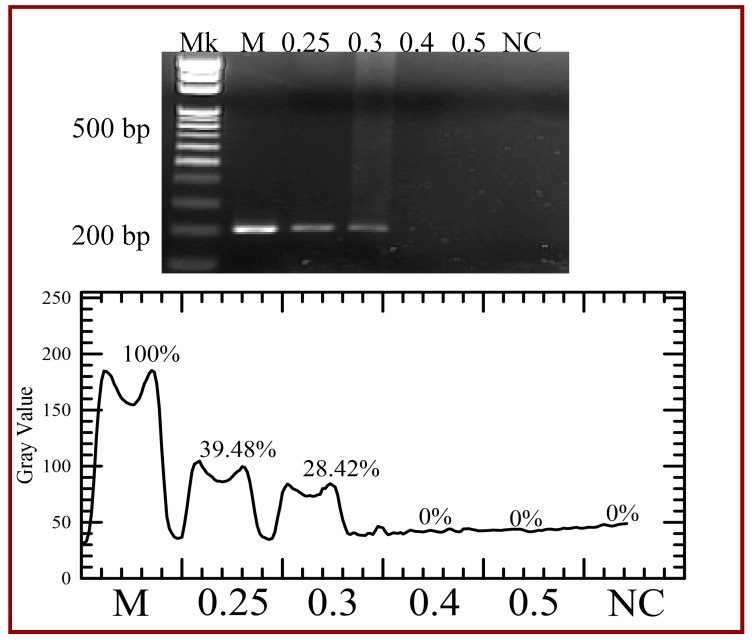
Agarose gel electrophoresis of the polymerase chain reaction (PCR) yield with various flow rates (Lanes 0.25, 0.3, 0.4, and 5). The grey intensities of PCR products are created by image analysis.

Results shown at the top of Figure 12 demonstrate the PCR product in the commercial PCR machine (Lane M), in our device with various flow rates (Lanes 0.25, 0.3, 0.4 and 5), and the DNA template for a negative control (Lane NC). The bottom of Figure 12 also illustrates the grey intensities of PCR products. As the flow rate increases, all the reaction times in each step become short and the time for amplification is insufficient. The reaction time can be calculated by the channel volume of the working region divided by the flow rate. So the cycle time associated with the flow rate of 0.2, 0.25, 0.3, 0.4, 0.5, 1, 5, 10 and 50 μL/min is 82.3 s, 65.8 s, 54.9 s, 41.1 s, 32.9 s, 16.5 s, 3.3 s, 1.6 s and 0.3 s, respectively. From Figure 9, [*D*] after 30 cycles is only about 100-fold more than that after 1 cycle with the flow rate of 1 μL/min. When the flow rate is larger than 5 μL/min, no longer does [*D*] almost increase after the cycle numbers larger than 10. Thus the sample flow rates utilized in the PCR are less than 0.5 μL/min.

**Figure 13:**
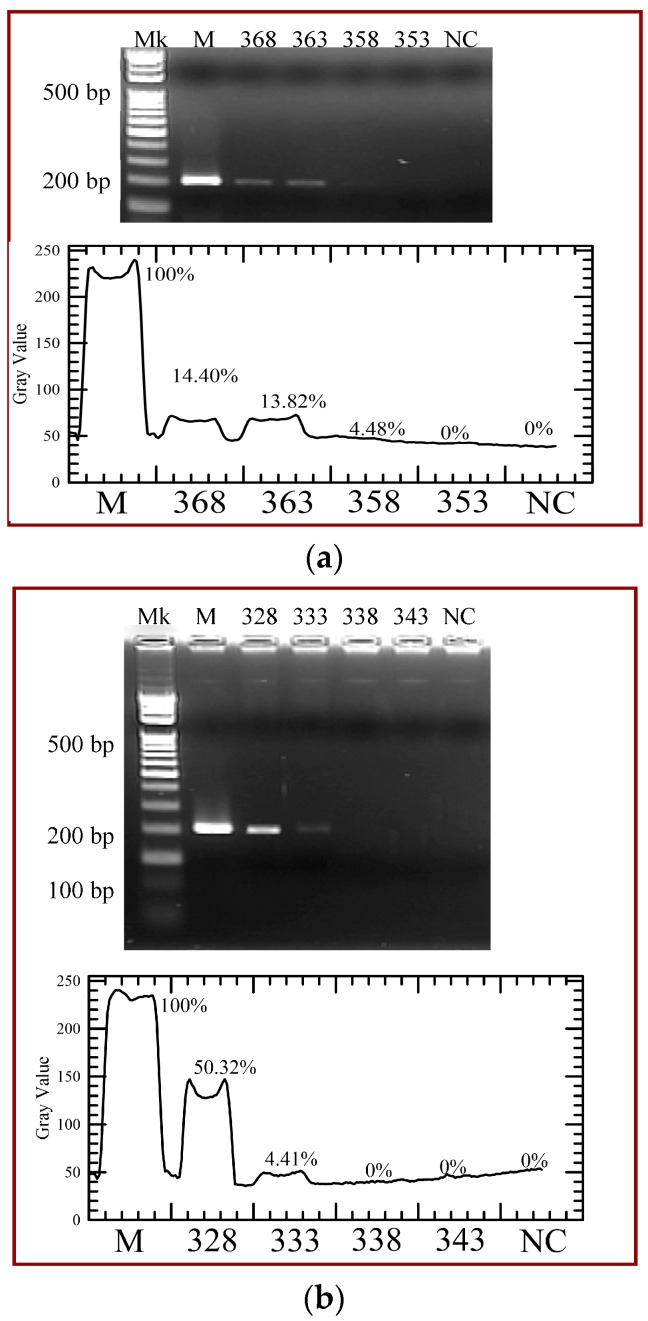
Agarose gel electrophoresis of the polymerase chain reaction (PCR) yield with (**a**) various heater temperatures for 368 K, 363 K, 358 K and 353 K and (**b**) various temperatures of the Peltier element for 328 K, 333 K, 338 K, and 343 K. The gray intensities of PCR products are created by image analysis.

The CFPCR chip is performed using various heater temperatures for 368 K, 363 K, 358 K and 353 K, and various temperatures of the Peltier element for 328 K, 333 K, 338 K and 343 K. Figure 13 shows the effects of various temperatures of the heater and the Peltier element on PCR amplification when the PCR mixture flows through the microchannel at the flow rate of 0.3 μL/min. It demonstrates that the amount of CFPCR products almost decreases with heater temperature from 368 K to 353 K, shown in Figure 13a. PCR performed with various temperatures of the Peltier element is shown in Figure 13b. When the temperature of the Peltier element increases, the PCR amplification time is decreased. For a successful PCR, the temperature of the DNA mixture should reach the requested temperature for each PCR step. In our simulated study, the melting temperature for specific DNA strands and the melting temperature of a primer are set to be 358 K and 338 K. The melting temperature for specific DNA strands is related to the length of the DNA molecule and its specific nucleotide sequence; the melting temperature of a primer also depends on both primer length and sequence. From our simulation results in DNA kinetic characteristics, the rate constants in denaturation and annealing steps have a very sharp transition where the rates change from minimum (maximum) values to maximum (minimum) values over a 10-degree temperature difference. The kinetic reactions happen if the mixture temperature reaches the temperature range in our simulated results. However, the trends of the experimental results and numerical data are similar.

## 5. Conclusions

In order to analyze the DNA amplification numerically with integration of the DNA kinetics, three-dimensional simulations, including flow and thermal fields, and one-dimensional PCR kinetics are presented. The performance of the CFPCR device fabricated on a PDMS-glass bonding chip with integrated a cartridge heater and a Peltier element is investigated by simulations and experiments. The governing equations, which consist of conservation of mass, momentum, and energy equations, are utilized to solve the flow and thermal fields in the CFPCR chip. An IR camera is used to characterize the spatial temperature distribution across the surface of the PDMS-based chip; the channel temperatures are measured by thermocouples that are inserted into the chip and put in contact with the glass. Furthermore, a visualization of the channel temperature distribution can be achieved by means of the thermally sensitive dye with an approximate transition temperature of 343 K. Numerical and experimental results have shown that the temperature distribution in the five-temperature-region PCR chip can be suitable for DNA amplification. The DNA kinetics in the CFPCR device is used to evaluate the DNA amplification by introducing the velocity and temperature profiles of the DNA mixture along the microchannel. Results show that for the mixture with a flow rate less than 5 μL/min, the heat convective effect inside the microchannel can be neglected and the temperature distributions for various flow rates are similar. However, the effect of the flow rates on [*D*] is noticeably more pronounced than the effect on temperature distribution. As the flow rate decreases, all the reaction times in each step become enough and the increasing of [*D*] is obvious. Furthermore, by the variation of the temperatures of the heater or the Peltier element, the resultant temperatures of the DNA mixture during the PCR steps are changed. The rate constants in denaturation and annealing steps have a very sharp transition where the rates change from minimum (maximum) values to maximum (minimum) values over a 10-degree temperature difference. The kinetic reactions can be happened ideally if the mixture temperature reaches within the temperature range. When the heater temperature is increased to be greater than 358 K (i.e., the melting temperature of double-stranded DNA molecules), the enhancement of [*D*] in the extension region is presented. As the temperature of the Peltier element equals 338 K (i.e., the melting temperature of a primer), binding between *S* and *P* is not enough during the annealing step and the DNA amplification is not very successful. When the temperature of the Peltier element is 343 K and almost no DNA amplification can be seen. The fabricated microfluidic chip is successfully capable of a 190-bp segment of *Bartonella* DNA amplification. The agarose gel electrophoreses of the PCR yields at various operational parameters are in similar trends with the simulation results. The major goals of this paper are to investigate the physical insights of the kinetic characteristics in the CFPCR device. Our future work is to optimize the geometry design and the operational parameters by systematically integrating a specific set of reaction parameters in the kinetic equations for the target DNA fragment. Future work will include a quantitative result to compare the predicted and measured PCR efficiencies to perform a comprehensive analysis of the unidirectional continuous-flow PCR devices.

## Figures and Tables

**Table 1:** Reaction parameters in the kinetic equations.

Parameter	Value
*k_D_*	2500L×12[1+tanh(T−3582)](s)−1
*k_−D_*	$10^{6}\times \frac{1}{2}\left[1+\tanh\left(-\frac{T-358}{2}\right)\right]{\left(\mathrm{mol}\cdot s\right)}^{-1}$
*k_SP_*	$10^{-4}\times \frac{1}{2}\left[1+\tanh\left(\frac{T-338}{2}\right)\right]{\left(s\right)}^{-1}$
*k_−SP_*	$5\times 10^{5}\times \frac{1}{2}\left[1+\tanh\left(-\frac{T-338}{2}\right)\right]{\left(\mathrm{mol}\cdot s\right)}^{-1}$
*k_e_*	$10^{8}\times \frac{1}{2}\left[1+\tanh\left(\frac{T-348}{2}\right)\right]{\left(\mathrm{mol}\cdot s\right)}^{-1}$
*k_−e_*	$10\times \frac{1}{2}\left[1+\tanh\left(-\frac{T-348}{2}\right)\right]{\left(s\right)}^{-1}$
*k_cat_*	$60\times e^{-{\left(\frac{T-348}{5}\right)}^{2}}{\left(s\right)}^{-1}$
*k_E_*	$1.9\times 10^{-4}\times \frac{1}{2}\left[1+\tanh\left(\frac{T-358}{2}\right)\right]{\left(s\right)}^{-1}$

**Table 2:** Initial concentrations of the species of the polymerase chain reaction (PCR) mixture.

Species	Initial Concentration (mol/L)
[*D*]_0_	0
[*S*]_0_	6.64 × 10^−15^
[*P*]_0_	2.23 × 10^−7^
[*SP*]_0_	0
[*E‧SP*]_0_	0
[*E*]_0_	4.95 × 10^−9^
